# An educator framework for organizing Wikipedia editathons for computational biology

**DOI:** 10.1093/bioinformatics/btaf216

**Published:** 2025-07-15

**Authors:** Nelly Sélem-Mojica, Tiago Lubiana, Toni Hermoso Pulido, Aarón Gallego-Crespo, Tülay Karakulak, Megha Hegde, Nicolas C Näpflin, Audra Anjum, Pradeep Eranti, Dan DeBlasio, Jorge Noé García-Chávez, Cynthia Paola Rangel-Chávez, Divanery Rodriguez-Gomez, Varinia López-Ramírez, Juan Vázquez-Martínez, Lonnie R Welch, Alastair M Kilpatrick, Farzana Rahman

**Affiliations:** Centro de Ciencias Matemáticas, Universidad Nacional Autónoma de México, Morelia 58089, México; Department of Genetics and Evolutionary Biology, Institute of Biosciences, University of São Paulo, São Paulo 05508-090, Brazil; Centre for Genomic Regulation, Barcelona Institute of Science and Technology, Barcelona 08003, Spain; Department of Hematology and Oncology, University Medical Center of the Johannes Gutenberg University, Mainz 55131, Germany; Department of Molecular Life Sciences and Swiss Institute of Bioinformatics, University of Zurich, Zurich 8057, Switzerland; School of Computing and Mathematics, Faculty of Engineering, Computing, and Environment, Kingston University, London, KT1 2EE, United Kingdom; Department of Molecular Life Sciences and Swiss Institute of Bioinformatics, University of Zurich, Zurich 8057, Switzerland; Office of Instructional Design, Ohio University, Athens, OH, 45701, United States; Université Paris Cité, Inserm, T3S, Paris 75006, France; Ray and Stephanie Lane Computational Biology Department, Carnegie Mellon University, Pittsburgh, PA, 15213, United States; Laboratory of Agrogenomics Sciences, Universidad Nacional Autónoma de México, León 37684, México; Biochemical Engineering Division, Tecnológico Nacional de México/Instituto Tecnológico Superior de de Irapuato, Guanajuato 36821, México; Biochemical Engineering Division, Tecnológico Nacional de México/Instituto Tecnológico Superior de de Irapuato, Guanajuato 36821, México; Biochemical Engineering Division, Tecnológico Nacional de México/Instituto Tecnológico Superior de de Irapuato, Guanajuato 36821, México; Chemical Engineering Division, Tecnológico Nacional de México/Instituto Tecnológico Superior de Irapuato, Guanajuato, 36821, México; School of Electrical Engineering and Computer Science, Ohio University, Athens, OH, 45701, United States; Centre for Regenerative Medicine, Institute for Regeneration and Repair, The University of Edinburgh, Edinburgh, EH16 4UU, United Kingdom; School of Computing and Mathematics, Faculty of Engineering, Computing, and Environment, Kingston University, London, KT1 2EE, United Kingdom

## Abstract

**Motivation:**

Wikipedia is a vital open educational resource in computational biology; however, a significant knowledge gap exists between English and non-English Wikipedias. Reducing this knowledge gap via intensive editing events, or “editathons,” would be beneficial in reducing language barriers that disadvantage learners whose native language is not English. Results: We present a framework to guide educators in organizing editathons for learners to improve and create relevant Wikipedia articles. As a case study, we present the results of an editathon held at the 2024 ISCB Latin America conference, in which ten new articles were created for the Spanish-language edition of Wikipedia. We also present a web tool, “compbio-on-wiki,” which identifies relevant English Wikipedia articles missing in other languages. We demonstrate the value of editathons to expand the accessibility and visibility of computational biology content in multiple languages.

**Availability and implementation:**

Source code for the compbio-on-wiki Toolforge site is available at: https://github.com/lubianat/compbio-on-wiki

## 1 Introduction

The online encyclopedia Wikipedia is one of the most frequently visited websites in the world. As an open education resource (OER), defined as a resource offering no-cost access, use, adaptation and redistribution of educational materials (Miao *et al.* 2016), Wikipedia is the most widely accessed in the field of computational biology (Kilpatrick *et al.* 2022). In a fast-paced field such as computational biology, OERs such as Wikipedia are well-placed to provide up-to-date information. For example, since the Pageviews analysis tool (https://pageviews.wmcloud.org) was released in July 2015, the English-language Wikipedia article “Bioinformatics” has been viewed over 1000 times a day on average; the “CRISPR” article has been viewed nearly 3000 times a day. The Computational Biology taskforce of WikiProject Molecular Biology (formerly WikiProject Computational Biology), a group of editors with expertise in computational biology and bioinformatics, has contributed content to English Wikipedia since its inception in 2007 (O’Neill *et al.* 2017). The taskforce aims to organize and improve articles relating to computational biology, of which there are around 1700.

In addition to English Wikipedia, as of December 2024 the non-profit Wikimedia Foundation operates an additional 339 Wikipedias in non-English languages. These Wikipedias provide an essential resource for learners whose native language is not English; however, a significant “knowledge gap” between computational biology coverage in English and non-English Wikipedias has been identified (Sélem-Mojica *et al.* 2024). As of December 2024, around 44% of English Wikipedia computational biology articles have no corresponding article in any non-English Wikipedia, a reduction of 3% from mid 2022 (Kilpatrick *et al.* 2022). This reduction is, at least in part, due to initiatives such as the annual Student Wikipedia Competition organized by the International Society of Computational Biology (ISCB) (Bateman *et al.* 2013, Kilpatrick 2016), which in recent years has offered a separate prize track for writing new articles and improving existing articles in languages other than English.

Recent studies have discussed the use of targeted Wikipedia editing events, or “editathons” (a portmanteau of “editing” and “marathon”), as pedagogical environments, framing Wikipedia as a learning tool, as well as an educational resource (March and Dasgupta, 2020, Littlejohn *et al.* 2021). Editathons are intensive editing events in the same tradition as “hackathons” in the technology community, where people engage in highly collaborative engineering over a short space of time. Situated Learning theory posits that learning occurs as a result of participation in communities of practice (Lave and Wenger 1991). As such, Wikipedia editathons are ideal environments for fostering situated learning through the process of legitimate peripheral participation, wherein a novice initially engages with a community of practice on the peripheral and gradually increases their participation. Editathons are designed to support entry into the Wikipedia editing community for novices so that they can participate in the co-construction and dissemination of knowledge.

There is a growing trend to incorporate Wikipedia-based writing activities within classroom settings (Kenny *et al.* 2013, Kilpatrick *et al.* 2020), following an “authentic learning” pedagogical model (Forte and Bruckman 2006). Editathons are typically small-scale events organized by volunteers, with a focus on specific topics, often with training and support for new editors. The educational value of editathons is apparent at several different levels. At the most basic level, editathons can improve Wikipedia as an OER, with editors improving existing articles and creating new articles; e.g. we have previously demonstrated editathons in non-English languages to quantifiably improve available educational resources relating to computational biology, specifically in Spanish-language Wikipedia (*Wikipedia en español*, henceforth “Spanish Wikipedia”) (Sélem-Mojica *et al.* 2024). Beyond this contribution to publicly-available knowledge, we have also previously highlighted Wikipedia editing as promoting editor’s learning via the “writing-to-learn” pedagogical approach, which focuses on deepening trainee understanding and concept retention via writing activities (Reynolds *et al.* 2012, Kilpatrick *et al.* 2020). More generally, Littlejohn *et al.* (2021) have described how editathons as a learning environment promote editors’ conceptual knowledge (the “what’s” and “why’s” of research, which need not be domain-specific, or even Wikipedia-specific) and procedural knowledge (how to effectively put these concepts into action through contributing to Wikipedia articles). The educational value of Wikipedia editathons for trainees thus aligns with several of the ISCB’s core competencies for computational biology, which have been iteratively developed over the past decade (Welch *et al.* 2014, 2016, Mulder *et al.* 2018, Brooksbank *et al.* 2024), most notably competency K3 (“communicate meaningfully with a range of audiences”) and M3 (“engage in continuing professional development in bioinformatics”).

Increasing the number and quality of non-English Wikipedia articles is important for learners who utilize Wikipedia as an OER. We previously highlighted knowledge gap issues related to equitable learning and cultural relevance (Sélem-Mojica *et al.* 2024). Having access to resources in one’s native or preferred language may reduce cognitive load, even for Wikipedia readers who speak English as a second language (Roussel *et al.* 2017, 2022).

We also highlight professional benefits to editathon organizers and facilitators (Osadebe *et al.* 2022). Again, these align with ISCB core competencies K3 and M3, and also with the leadership elements of competency L3 (“work effectively in teams to accomplish a common goal”) (Brooksbank *et al.* 2024).

Given the knowledge gap in topics relating to computational biology between English and non-English Wikipedias, and the potential for this to be narrowed by targeted editing events, in this study we present a framework for computational biology educators seeking to organize a successful Wikipedia editathon, using the ISCB Latin America (ISCB-LATAM) Wikipedia Editathon and the contributions of its participants to Spanish Wikipedia as a case study. The recommendations presented here are primarily based on qualitative observations and the experience gained during the implementation of editathons, as well as feedback collected from participants, facilitators, and organizers since the inception of the ISCB Wikipedia Competition. While no formal statistical analysis was conducted, we aimed to reflect the practical insights derived from the process to propose actionable recommendations. We describe the development of a web-based tool for discovering knowledge gaps in computational biology coverage in non-English Wikipedias, then highlight its use in the wider context of organizing and running a successful small-scale editathon. We demonstrate the value of the ISCB-LATAM Wikipedia Editathon in improving computational biology coverage in Spanish Wikipedia. Finally, we discuss areas for further refinement and highlight initiatives for continuing this work.

## 2 Materials and methods

### 2.1 Article definition and metadata

English Wikipedia articles relating to computational biology were defined as described previously (Kilpatrick *et al.* 2022). Briefly, these are articles tagged by Wikipedia editors as being within the scope of the Computational Biology taskforce of WikiProject Molecular Biology. Article page sizes at specific times were retrieved from each article’s history page, which tracks an article’s edits over time. Page views for the first 30 days following article creation were obtained using the xtools website (https://xtools.wmcloud.org).

### 2.2 Article content assessment

English Wikipedia articles are rated for importance, or relevance, for each WikiProject by Wikipedia editors. As described previously, article importance is rated on a four-point scale increasing through Low, Mid, High and Top importance (Kilpatrick *et al.* 2022). Importance ratings for the Computational Biology taskforce of WikiProject Molecular Biology were used in this study, again assuming that an article’s relevance to computational biology is independent of language.

Similarly, Wikipedia articles are rated for quality by Wikipedia editors according to site-wide defined quality criteria, as described previously (Sélem-Mojica *et al.* 2024). Briefly, articles are rated on an increasing scale from Stub class (lowest quality) to the peer-reviewed Good Article (GA) and Featured Article (FA) classes. Spanish Wikipedia does not have similar site-wide quality assessment criteria, but WikiProjects may define their own article quality classes. As in previous studies, here we used the ratings suggested by Spanish WikiProject Cellular and Molecular Biology (*Wikiproyecto Biología celular y molecular*); articles of increasing quality progress from *Esbozo* (E; “outline”), through *Artículo poco Desarrollado* (ApD; “underdeveloped article”), *Artículo* (A; “article”), and *Artículo Bueno* (AB; “good article”) to *Artículo destacado* (AD; “featured article”) classes (Sélem-Mojica *et al.* 2024).

### 2.3 The compbio-on-wiki web tool

The compbio-on-wiki web tool uses Flask, a Python web framework (Grinberg 2018) and is hosted on Toolforge, a cloud service provided by Wikimedia for community projects. The tool integrates the SPARQL queries and pre-run calls to MediaWiki Actions API of English Wikipedia to create a user-friendly interface that lists computational biology articles missing in a selected target language. The Python code uses SPARQLWrapper (v2.0.0) to remotely execute SPARQL queries and pandas [v2.3.3; McKinney (2010)] to load Wikidata IDs for relevant English Wikipedia articles. The source code is publicly available at: https://github.com/lubianat/compbio-on-wiki.

## 3 Results

### 3.1 A framework for editathon organisation

**Figure 1.**
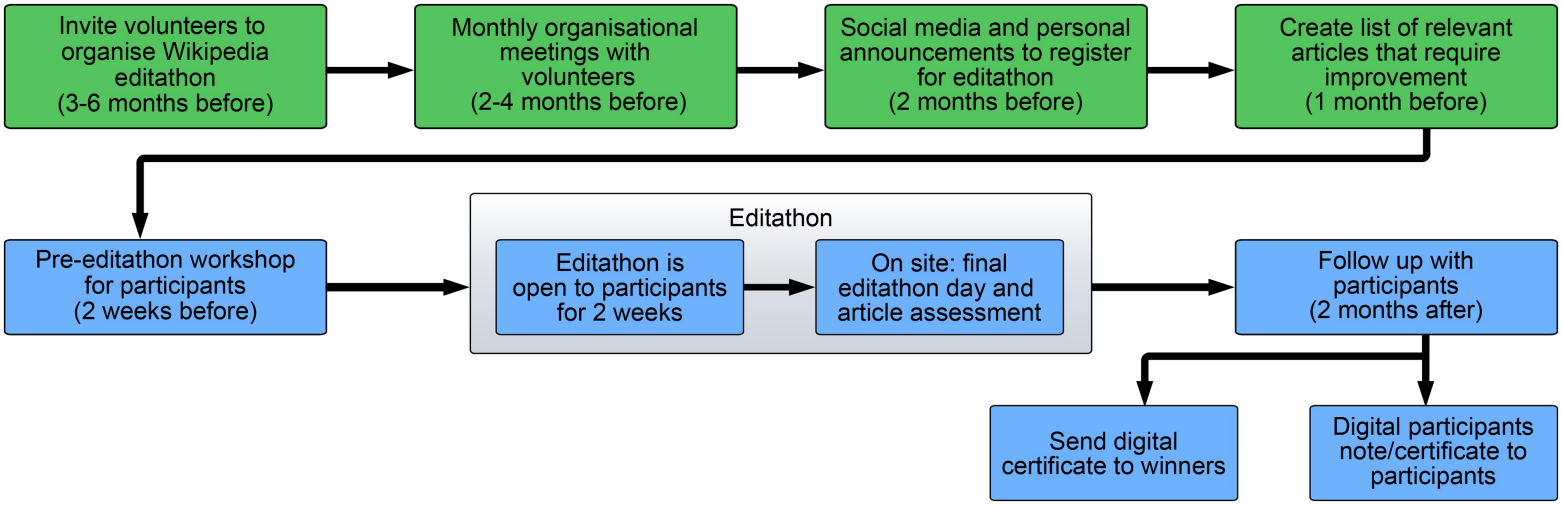
The organizational structure of the editathon is illustrated, with suggested timescales in reference to the editathon event. Processes for organizers are in green; processes with editathon participants are in blue.

Our organizational framework for educators wishing to host a Wikipedia editathon is presented in Fig. 1. While editathon participants edited Wikipedia for between 1 and 15 days, significant preparation time in advance of the event is required to ensure a successful outcome.

We recommend inviting volunteers to organize the editathon 3 months in advance. Volunteers typically take on one of three roles: editing facilitators, subject matter experts, or contest communicators. Ideally, having at least two volunteers per role ensures smooth coordination and keeps the workload manageable (we estimate 2–3 h per week). The tasks of *editing facilitators* include preparing and delivering the introductory workshop and assisting participants with practical questions during the editathon. *Subject matter experts* are responsible for selecting a list of relevant topics or articles for the event. If there is a competitive element to the editathon, they may also serve as judges, or help to identify appropriate judges for the articles. *Communicators* are tasked with inviting their collaborators to participate in the contest and promoting the event on social media. Required skills for volunteers depend on their role. For instance, editing facilitators should have extensive experience as Wikipedia editors, whereas subject matter experts and communicators need not necessarily know how to edit Wikipedia. Instead, their ability to attract and engage potential participants is more critical to fulfilling their roles. Roles are not mutually exclusive; e.g. subject matter experts may also be editing facilitators.

Once a volunteer team has been recruited, we recommend monthly team meetings to prepare a welcome workshop and registration forms for participants. If the editathon is to include a competitive element, the volunteer team will also have to prepare an assessment rubric and forms for judges to assess the changes made to the edited articles. Although full committee meetings can be limited to once a month, communication should remain ongoing either by email or social media channels. We recommend creating a shared collaborative document from the outset that is accessible to all and where short-, medium-, and long-term goals are outlined. This document can be used to assign tasks to volunteers or allow each volunteer to propose how they can contribute to the event. This approach enables members with similar organizational roles to meet separately and track progress before the full committee meeting. These full committee meetings provide an opportunity to review progress, update the working document, and reassess strategies for achieving upcoming short-term goals.

In our experience, offering formal recognition improves participation in Wikipedia editing events. Crafting a Wikipedia article requires several skills, including researching the topic, organizing information, citing sources, creating visual aids to clearly and concisely explain concepts, and writing in a neutral tone. These skills deserve credit; we recommend awarding all editors a certificate of participation. Monetary awards for the most impactful contributions will draw increased participation; recognition for these awards can enhance participants’ resumes and help them achieve their personal goals.

We suggest communicators coordinate publicity and logistics with relevant strategic partners. Examples may include professional societies’ mailing lists, educators, past participants, Wikimedia and student groups. One to 2 months of outreach including at least 1 month of social media promotion and personal invitations is suggested to engage with the academic community and allow registration time.

To effectively focus the work of editathon participants, it is important that subject-matter experts compile a list of relevant and impactful articles to work on. This may be based on emerging topics that are not yet reflected on Wikipedia; another option may be to focus on improving underdeveloped articles. For editathons on non-English Wikipedias, this list could be derived from the number of visits to English articles that are missing or underdeveloped in the language of choice. Subject-matter experts may collaborate with editing facilitators to create an interactive list using Wikipedia tools that track metrics such as page views by topic or article size.

We strongly recommend a pre-event workshop, held as a virtual meeting, around 2 weeks before the in-person editathon. Participants should be guided on creating a Wikipedia account, briefed on the core principles of Wikipedia editing, and introduced to the list of recommended articles. They should also be informed that Wikipedia facilitator volunteers will be available to assist with practical editing questions during the editing period. For new articles, participants should be advised to ensure their drafts are well-developed (with multiple sections and at least 10 references) before uploading them to Wikipedia, to minimize the risk of deletion. They should also be reminded that a diverse community edits Wikipedia, so discussions about an article’s relevance or writing quality may arise as a normal part of the collaborative editing process. Following the workshop, participants will have an editing period of around 2 weeks to work on their chosen articles. They may wish to work in teams and seek assistance from editing facilitator volunteers as required. Some teams may have external reviewers, such as subject-matter experts or university professors. Teams or individual participants may create and upload the content to Wikipedia at any point during this period. Since making a new article and becoming familiar with Wikipedia’s editing process can take time, we recommend dedicating the first week to research, starting the editing on Wikipedia at least 1 week before the in-person editathon.

The in-person editathon event, which may be associated with a larger professional society gathering, represents the final day of the editathon. For participants who attended the pre-event workshop, this final day allows them to finalize their articles and resolve any last-minute questions with their peers and the organizers. Participants who are only joining in person should be invited to attend a condensed version of the introductory workshop, select their articles, and make initial edits to get involved with the editing process. From the organizers’ perspective, the event may span up to 3 days: the first and main day is dedicated to supporting participants at the in-person editathon event. For editathons with a competitive element, we suggest an additional 2 days for the judges to conduct their assessments and to announce the winners. The judges required will depend on the number of participants and articles in the editathon. We suggest each judge assesses 3–5 articles, with each article assessed 2–3 times to minimize bias. The winner may be chosen based on the highest average score, with ties allowed. The Wikipedia content assessment guidelines (https://en.wikipedia.org/wiki/Wikipedia: Content_assessment) may be useful in constructing an assessment rubric (Kilpatrick *et al.* 2022).

After the editathon, following up with participants to encourage their longer-term interest in Wikipedia editing is vital. For participants choosing to continue to improve their articles, providing feedback soon after the editathon based on the assessment rubric is useful. In addition to feedback, issuing participation certificates and recognitions can further encourage participant engagement.

### 3.2 Case study: ISCB-LATAM Wikipedia editathon

As part of its mission, the ISCB Wikipedia Committee aims to organize activities that ignite academic interest and foster long-term participation in editing Wikipedia articles relating to computational biology. In alignment with this goal, the committee hosted an editathon to promote academic community involvement in the 14th ISCB Student Wikipedia Competition, which runs from September 2024 to May 2025. The ISCB Latin America (ISCB-LATAM) Wikipedia editathon ran from 30 October to 14 November 2024 and aimed to enhance Computational Biology articles in non-English Wikipedias, primarily Spanish and Portuguese. While we present a case study on editing computational biology articles in Spanish, the experience is broadly applicable to editathons centered on academic or specialized content.

An eight-member volunteer committee began organizational preparations 3 months before the event. The team included three editing facilitators, four communicators, and a general organizer, all actively working in the field of computational biology. The event was promoted via social media, academic conferences, via email to ISCB Regional Student Groups (RSGs) and ISCB members.

**Figure 2.**
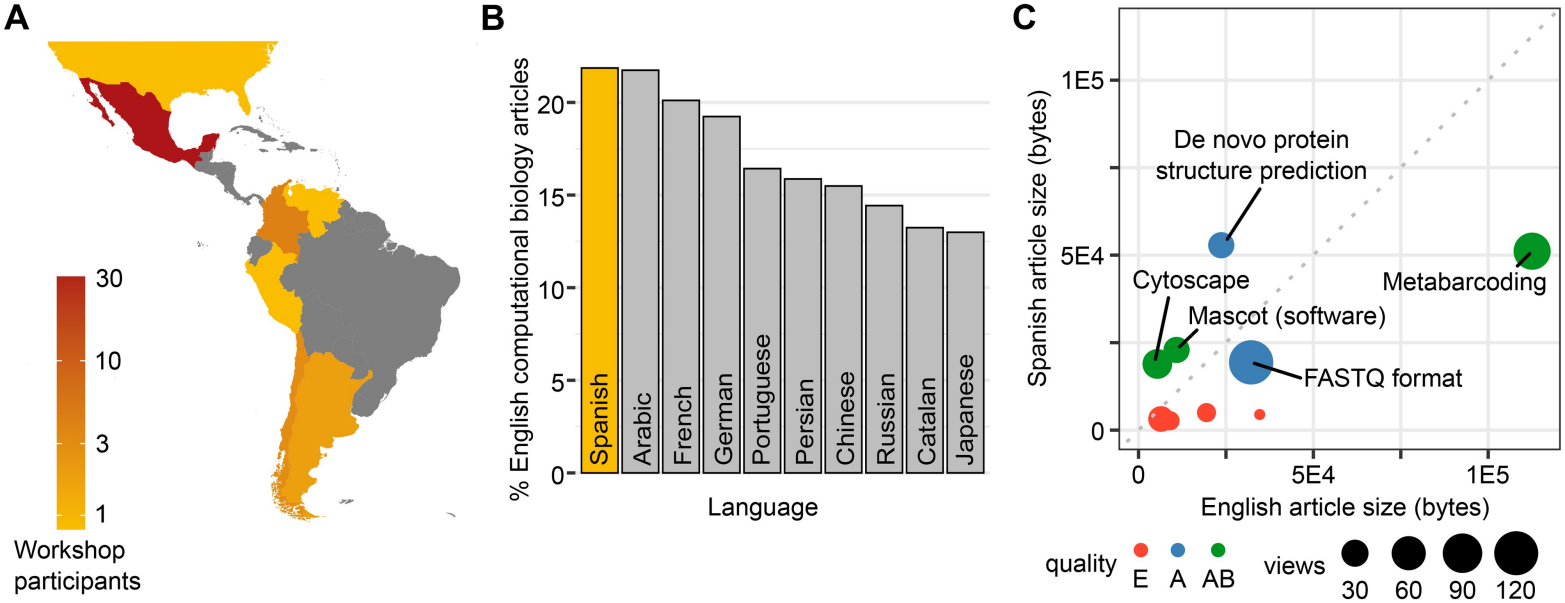
ISCB-LATAM editathon impact on Spanish Wikipedia. (A) Map showing participation from Latin and South American countries. Grey: no participants. (B) Following the editathon, Spanish Wikipedia has the highest number of computational biology articles, surpassing Arabic Wikipedia. (C) Scatterplot of articles created in the ISCB-LATAM editathon, comparing article size in English Wikipedia (*x*-axis) to that in Spanish Wikipedia (*y*-axis). Circle size represents the number of views each article received over the last 30 days. Color represents article quality class: E (orange); A (blue); AB (green).

For participants, the editathon began with a 1-day virtual Wikipedia workshop designed and led by volunteer editing facilitators. The workshop content was based on volunteers’ prior experiences, including two workshops supporting the annual ISCB Student Wikipedia Competition and collaborations with Wikimedia México. In this initial workshop, participants were shown how to create a Wikipedia account, make their first edit, and understand the “Ten Simple Rules” for editing Wikipedia (Logan *et al.* 2010). One of the most important of these rules is learning the “Five Pillars,” Wikipedia’s fundamental principles; these include points such as Wikipedia being a free-content encyclopedia, and that editors should strive for a neutral point of view. A list of articles relating to computational biology was also presented to participants. Fifty-four participants registered for the workshop; 76% represented Latin America, with the majority from Mexico (Fig. 2A). The rest registered from the USA, Europe, and India. Over the next 2 weeks, participants worked on editing articles either individually or in teams, supported by the editing facilitators as required. 54% of participants registered as part of a team; 46% participated individually. Among the teams with the most significant progress, two were graduate students working independently, while three were senior undergraduate students guided by their professors. The university professors acted as external advisors, providing corrections and suggestions for edits without directly editing the articles themselves.

The in-person editathon event was held during the ISCB-Latin America SoIBio CCBCOL Conference on Bioinformatics (ISCB-LATAM) at Universidad CES, Medellín, Colombia, in November 2024. Personal invitations were extended to attendees during the conference. For new participants, a shortened version of the workshop was offered, focusing on presenting the list of relevant articles and assisting them with their first edit. New participants had 1 day to continue editing. Following the editing period, three judges assessed the articles that had progressed beyond *Esbozo* (stub) quality using a rubric. The event concluded with an awards ceremony and optional feedback sessions for participants who requested it. Certificates of participation were distributed. Participants were encouraged by a post-editathon email to continue editing their articles and to consider entering them into the ISCB Student Wikipedia Competition.

During the 2-week editing period, 54 participants claimed 21 Wikipedia articles to work on. By the end of the period, 10 articles (all on Spanish Wikipedia) had been edited by a total of 21 users (Table 1). Eleven claimed articles remained unedited; participants did not attend follow-up meetings.

**Table 1.** Articles edited in the ISCB-LATAM Wikipedia editathon.^a^

Article title (Spanish)	Article title (English)	Spanish article quality	English article size (bytes)	Spanish article size (bytes)	30-day views	Number of participants
Metabarcoding	Metabarcoding	AB	112 557	51 130	76	4
Cytoscape	Cytoscape	AB	5383	18 907	40	3
Mascot	Mascot (software)	AB	10 880	22 909	27	3
*Predicción de la estructura*	*De novo* protein					
*de proteínas de novo*	Structure prediction	A	23 657	52 833	27	3
*Formato FASTQ*	FASTQ format	A	32 197	19 371	122	3
*Algoritmo de Kabsch*	Kabsch algorithm	E	9017	2682	9	1
DAVID	DAVID	E	6418	3185	25	1
*Penalización por espacio*	Gap penalty	E	19 431	5007	9	1
Virtual screening	Virtual screening	E	34 651	4457	2	1
*Lista de nociones de forcing*	List of forcing notions		16 640			1

a For each article, the Spanish Wikipedia quality ratings following the editathon, the English and Spanish page sizes (in bytes), the number of page views in the 30 days following the editathon and the number of editing participants are provided. *Lista de nociones de forcing* was subsequently deleted from Spanish Wikipedia by other users. Spanish article quality ratings: E, *Esbozo* (“outline”); A, *Artículo* (“article”); AB, *Artículo Bueno* (“good article”).

After the in-person event, the volunteer committee filtered out underdeveloped articles. Three judges assessed five articles each, with each article reviewed twice using a rubric developed for assessing entries in the ISCB Student Wikipedia Competition. Only two articles included primary references in Spanish. The highest-scoring articles were all edited by teams of at least three people. After the editathon, all articles were independently assessed for quality by one of the subject matter expert volunteers. Five of the articles were assessed as stubs (E class), two as articles (A), and three as good articles (AB).

Judges assessed clarity of writing, depth of content, inclusion of additional materials, and the presence of native-language references. The five most developed articles were *Metabarcoding*, *Cytoscape*, *Mascot*, *Predicción de la estructura de proteínas de novo*, and *Formato FASTQ*. While all the articles were primarily based on their English counterparts, each included unique content adapted to Spanish. The article *Predicción de la estructura de proteínas de novo* was the most developed, with a page size of 52 833 bytes after editing, followed closely by *Metabarcoding* with 51 130 bytes. The overall winning article was *Metabarcoding*, which averages 1800 monthly visits to English Wikipedia as of 2024, yet was not previously part of Spanish Wikipedia.

Although other articles excelled in specific aspects, *Metabarcoding* won the competition due to its consistently high performance across all three key rubric categories: depth of content, clarity of writing, and inclusion of additional materials tailored to the Spanish audience. Additionally, it featured well-curated references in Spanish, aligning with the requirements of the edited Wikipedia language. Its success may also be attributed to the fact that it closely mirrors the structure and quality of its English counterpart, which has existed since 2021 and has been extensively refined over time.

The editathon rubric (Table 2) was developed iteratively using a backward design approach (Wiggins and MacTighe 2005). A computational biology subject-matter expert (SME) and instructional designer (ID) examined exemplary Wikipedia articles within the computational biology domain. After determining evaluation categories that represent quality contributions (depth of content, clarity of writing, inclusion of additional materials), these articles were used as anchors for the different levels of the rubric categories. The SME wrote descriptors for each level of each category to delineate different levels of quality among the article enhancements. The ID ensured that the descriptors were written to be both analytic and holistic (Stevens and Levi 2023) enough to capture broad impact across different articles and subjects. The levels broadly match English Wikipedia’s content assessment scale, although there is more focus on pedagogical assessment.

**Table 2.** A rubric for assessment of edited Wikipedia articles.

Criteria	Incomplete	Poor	Satisfactory	Good	Excellent
Clarity of writing	Lacks focus, incompletely developed, few details.	Partially developed, lacks details, many grammar and punctuation errors.	Organized, supports the central idea. Few grammar or punctuation errors.	Well-structured, flows smoothly. No grammar or punctuation errors.	Focused, well-developed, detailed, and precise. Uses original, fresh words.
Depth of knowledge of subject area	No original contribution of material, or incorrect information.	Little or no evidence of critical, creative thought, analysis or insight.	Relevant definitions and explanations added to the article. Reliable scientific sources used. Source material reworded in own words.	Evidence of critical, creative thought, analysis or insight. The article may be enhanced with relevant examples or comparisons.	Abundant evidence of critical, creative thought or insight. Relevant examples, comparisons and discussion must be included.
Quality of illustrative figures, equations or pseudocode	No illustrative items added.	Minimal, irrelevant or low quality illustrative items added. Items not described in article.	Relevant illustrative items for some key concepts along with textual descriptions.	Relevant illustrative items for most key concepts along withtextual descriptions.	High quality illustrative items and accompanying text that provide substantial depth of insight for all key concepts.

All articles worked on during the ISCB-LATAM editathon (Table 1) were new articles, rather than edits of existing articles. These newly created articles have led to Spanish Wikipedia overtaking Arabic Wikipedia as the non-English Wikipedia with the highest coverage of computational biology, as measured by number of articles, as of December 2024. 350 English Wikipedia articles relating to computational biology now have Spanish language equivalents (21.9%), compared to 348 in Arabic Wikipedia (21.7%) (Fig. 2B). Comparing article sizes between Spanish and English Wikipedia (Fig. 2C) reveals that three articles (*Mascot*, *Cytoscape*, and *Predicción de la estructura de proteínas de novo*) have more content in Spanish Wikipedia than in English. Undergraduate teams created these articles. E class articles fall below 10 000 bytes in size and require further development.

The article with the most views in the first 30 days (122) was *Formato FASTQ*, assessed as A class for article quality (Table 1). The three articles rated AB for quality had an average of 47 views over the 30 days following the editathon; in contrast, the E class articles had an average of 11 views. Together, the articles created in the editathon were visited approximately 350 times in the first 30 days, highlighting their impact on the Spanish-speaking computational biology community.

### 3.3 Choosing impactful articles to edit

Given the breadth of the computational biology field, one important consideration is selecting articles to be edited. This may be guided by editathon organizers curating a list of articles that would be most impactful for the participants to work on.

A list of English Wikipedia articles tagged by users as being of relevance to the Computational Biology taskforce of WikiProject Molecular Biology is available https://wp1.openzim.org/#/project/Computational_Biology/articles. This list of around 1700 articles may be of use to editathon organizers in curating a shortlist of impactful articles. The list can also be sorted and filtered based on importance and quality.

**Figure 3.**
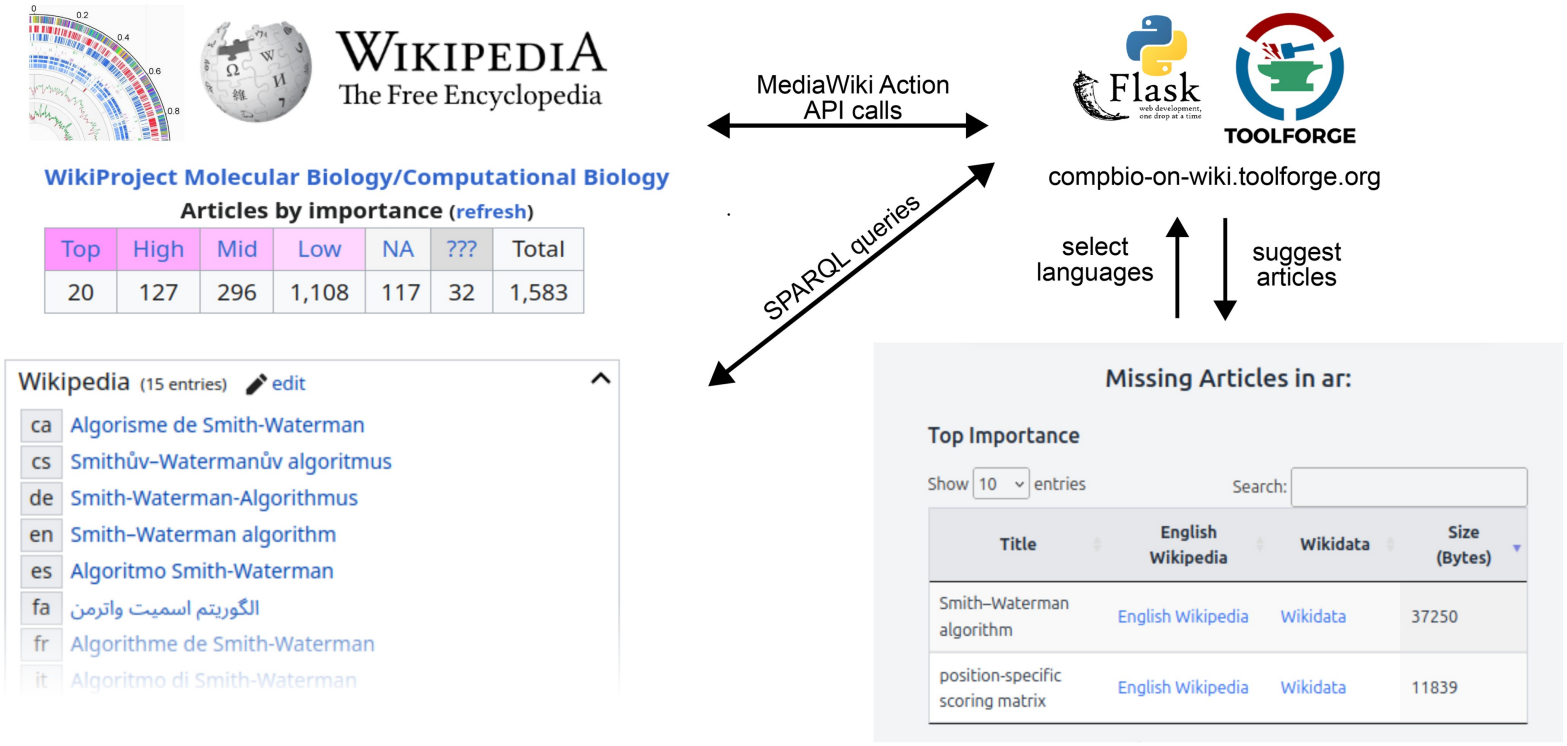
The compbio-on-wiki web tool helps participants select impactful articles to edit. The Flask application retrieves a list of computational biology articles from English Wikipedia and uses Wikidata to find links to other language versions. Articles present in English but missing in the user’s non-English language of choice are displayed.

To facilitate the identification of articles missing in non-English Wikipedia versions, we designed and implemented a web tool “compbio-on-wiki,” written using the Flask framework for Python and available at https://compbio-on-wiki.toolforge.org/. This web tool uses categories from relevant articles in English and identifies those articles lacking equivalent pages in other languages (Fig. 3). Wikidata (Vrandečić *et al.* 2023), a sister project of Wikipedia, holds the interlinks between Wikipedia language versions and thus the compbio-on-wiki tool can leverage this data to suggest articles relating to computational biology which are present in English Wikipedia and missing in non-English Wikipedias. Given a non-English language query, compbio-on-wiki retrieves a list of relevant articles from English Wikipedia via a MediaWiki Action API call. This data is then used in a SPARQL query to Wikidata, which returns the articles in the list which also appear in the query language. The data is returned to the user, ordered by the four-point importance scale for English language articles, allowing the user to select candidate articles for translation.

ISCB-LATAM Wikipedia editathon participants were introduced to the compbio-on-wiki tool and used it to select articles for editing, to ensure that they worked on relevant articles. While editathon participants used Spanish as the query language, the compbio-on-wiki tool is language-agnostic, and is able to suggest articles for all non-English Wikipedias, facilitating contributions in >300 other languages.

## 4 Discussion

There has recently been rapid growth in non-English Wikipedias, and we have previously discussed the importance of their presence as OERs for learners whose native language is not English (Sélem-Mojica *et al.* 2024). However, a significant knowledge gap has been demonstrated between these resources and English Wikipedia (Roy *et al.* 2022), and we have shown this for computational biology. Here, we demonstrate the positive impact that editathon events can have on non-English Wikipedias as OERs, using Spanish Wikipedia and the 2024 ISCB-LATAM editathon as a case study.

The articles most highly rated for quality in our editathon provide evidence for the benefits of co-authoring, or collaborative writing; we have observed similar trends in the most successful entrants to the ISCB Student Wikipedia Competition. While there is clearly an argument that suggests that more writers yield more text, we suggest that in this context it is also not a case of “too many cooks”: the act of collaborative writing also ultimately yields better quality text. Beyond the text, pedagogical research also suggests that collaborative writing is beneficial for students. When co-authoring, students function both as readers and writers; the process promotes both the cognitive and social aspects of writing (Dale 1996). Writing fluency has also been shown to improve (both in group and individual writing tasks) following collaborative writing exercises (Lopres *et al.* 2023). Writing as a group also furthers the teamwork elements of ISCB core competency L3 (Brooksbank *et al.* 2024).

Specific feedback from participants included: “Current and complex topics were addressed, with an emphasis on ensuring they were made readable and accessible.” An educator noted: “I believe that teaching is learning twice; students were able to refine how they shared their knowledge, which in turn strengthened the research topics they were working on.” Several participants highlighted the flexibility of the online format, which encouraged teamwork and provided greater opportunities to contribute to an OER. Students also expressed enjoyment in learning how to edit and structure Wikipedia articles for a general audience, gaining valuable skills in clearly explaining concepts in computational biology. The experience also strengthened participants’ collaboration skills and underscored the importance of effectively sharing knowledge, reinforcing their research expertise. Though many were accustomed to using Wikipedia as readers, editing posed challenges due to unfamiliarity with the platform’s rules, systems, and quality expectations. The editathon addressed these obstacles and opened new avenues for academic dissemination. The event fostered a new cohort of Wikipedia editors who, through guided support, gained confidence in their contributions. While Wikipedia’s editing guidelines are accessible online, personalized guidance from subject-matter experts, particularly in identifying relevant topics, provided participants with the confidence and assurance needed to contribute meaningfully.

The framework is a living document that may be iteratively improved. The experience of hosting the introductory workshop highlighted areas of potential improvement. This could have benefited from being less lecture-like and more hands-on to improve engagement. For example, after participants have registered and made their first edit, it may be beneficial to give them time to work through an online tutorial for beginners such as the Wikipedia Adventure (https://en.wikipedia.org/wiki/WP: TWA). Following the editathon, we designed an online guide with frequently asked questions (https://en.wikipedia.org/wiki/WP: ISCB-EDIT-FAQ). Many participants attempted to join the editathon workshop via smartphones rather than computers. Editing Wikipedia is possible on smartphones; however, in our experience, this is easier on a desktop or laptop computer. One improvement would be to note this in the advertising communications.

Future iterations will also highlight the benefits of starting Wikipedia articles in user “sandboxes.” These are semi-private areas within a user’s Wikipedia profile where articles may be drafted before they are moved to the main Wikipedia user space and may be edited, or even deleted, by other Wikipedia users (Kenny *et al.* 2013). The editathon article *Lista de nociones de forcing*, deleted by another Wikipedia user as being “irrelevant” despite the corresponding article being available on English Wikipedia, may not have suffered this fate had it been developed further in this way.

We have previously discussed the difficulty in finding suitable references in languages other than English (Sélem-Mojica *et al.* 2024). This is at least in part due to publishing in English-language journals being more desirable given their historically higher impact and increased exposure potential. Throughout the editathon, we made sure to highlight the value of native-language citations; however, including a number of relevant English references is essential, given the literature in a relatively nascent field such as computational biology.

Machine translation and large language models (LLMs) have been proposed to aid bridge language barriers and accelerate expansion of non-English Wikipedias. However, automated translation may be limited by either poor direct translation or shallow template-based translation complicated further by the use of neologisms in computational biology and bioinformatics (Sélem-Mojica *et al.* 2024). While AI is used in some Wikipedia projects, including machine translation (Alshahrani *et al.* 2023), the Wikipedia community consensus as of March 2025 is to prefer human decisions over machine-generated outcomes until the implications of AI are better understood (https://en.wikipedia.org/wiki/WP: AI). In an educational setting, we note that many institutions now implement their own guidance and policies on the use of LLM-generated content; this often extends to scholarly societies, including the ISCB (https://www.iscb.org/iscb-policy-statements/iscb-policy-for-acceptable-use-of-large-language-models).

A limitation of the compbio-on-wiki tool is that it is based on English Wikipedia articles tagged manually by editors as relevant to computational biology. This set of tagged articles would benefit from regular updates to remain relevant as the field progresses. We additionally recognize that there may be unconscious biases within the set of tagged articles, due to the scientific interests of the tagging editors; we have previously noted the variability in coverage between different computational biology subfields (Kilpatrick *et al.* 2022). Semi-automated methods of tagging relevant articles based on the computational biology literature or keywords provided by ISCB communities of special interest may be useful in expanding the set of relevant articles.

While the ISCB-LATAM editathon prioritized editing articles in Spanish and Portuguese, the knowledge gap between English and non-English Wikipedias in computational biology is not limited to these languages and is generally broader in other languages. The pedagogical value of non-English OERs indicates a need to rapidly upscale efforts in all languages, and we call for similar initiatives, potentially in collaboration with the ISCB’s Regional Student Groups and affiliate groups, which operate worldwide. While there are many multilingual Wikipedia users, only around a quarter edit articles in multiple languages (Hale 2014); increasing this fraction by utilizing the collective knowledge of computational biology students could lead to a rapid growth in non-English educational resources.

Finally, in the future we aim to develop methods to incorporate editathons into classrooms where Wikipedia editing projects are already used. In this context, editathons will be used to help students learn about Wikipedia editing and to empower them to iteratively outline and draft high-quality article enhancements.

## 5 Conclusion

Wikipedia is the most widely used OER in computational biology. Wikipedia editathons have a unique role to play in educational settings, in developing participants’ core competencies while also providing a public service in improving this vital OER. Here, we presented the first iteration of a framework for educators wishing to organize editathons for articles relating to computational biology. Using Spanish Wikipedia and the ISCB-LATAM Wikipedia editathon as a case study, we quantitatively demonstrated the impact that these short, intensive editing events can have on non-English Wikipedias. Finally, we presented a new web tool to identify articles relevant to computational biology but missing from non-English Wikipedias, maximizing participant impact.

## Supplementary Material

btaf216_Supplementary_Data

## Code availability

Source code for the compbio-on-wiki Toolforge site is available at: https://github.com/lubianat/compbio-on-wiki
