# Supplementary material for: An educator framework for organizing Wikipedia editathons for computational biology
Source: Bioinformatics. 2025 Jul 15;41(Suppl 1):i12–20. doi: 10.1093/bioinformatics/btaf216 (PMC12261409; doi:10.1093/bioinformatics/btaf216)
Supplement: btaf216_Supplementary_Data [file btaf216_supplementary_data.docx]

Fig. 1.

Graphical representation of a timeline for editathon organization, highlighting major steps in the process for organizers and attendees.

Fig. 2.

Graphs and data on the impact of the ISCB-LATAM Wikipedia editathon on Spanish Wikipedia, with subfigures labelled from a to c, illustrating editathon participant locations and comparisons to Wikipedias in other languages.

Fig. 3.

Graphical representation of how the compbio-on-wiki tool works, illustrating the calls the tool makes to Wikipedia and Wikidata.

Table 1.

Table summarising the articles edited in the ISCB-LATAM Wikipedia editathon.

Table 2.

Table describing the rubric used in assessment of edited Wikipedia articles.

Keywords:

Education, Open education resources, Collaborative writing, Wikipedia, Spanish language

Nelly Sélem-Mojica nselem@matmor.unam.mx

Tiago Lubiana tiagolubiana@gmail.com

Toni Hermoso Pulido toni.hermoso@crg.eu
Aarón Gallego-Crespo aargalcre55@hotmail.com

Tülay Karakulak tulaykarakulak@gmail.com

Megha Hegde Megha.Hegde@kingston.ac.uk

Nicolas C. Näpflin nicolas.naepflin@mls.uzh.ch

Audra Anjum hilterbr@ohio.edu

Pradeep Eranti pradeep.eranti@gmail.com

Dan DeBlasio dan@dandeblasio.com

J. Noé García-Chávez j.noe.garcia.c@gmail.com

Cynthia Paola Rangel-Chávez cynthia.rc@irapuato.tecnm.mx

Divanery Rodriguez-Gomez divanery.rg@irapuato.tecnm.mx

Varinia López-Ramírez,

Juan Vázquez-Martínez

Lonnie R. Welch welch@ohio.edu

Alastair M. Kilpatrick alastair.kilpatrick@ed.ac.uk
Farzana Rahman farzana@kingston.ac.uk
